# Design and Characterization of a Membrane Protein Unfolding Platform in Lipid Bilayers

**DOI:** 10.1371/journal.pone.0120253

**Published:** 2015-03-23

**Authors:** Vincent G. Nadeau, Anqi Gao, Charles M. Deber

**Affiliations:** 1 Division of Molecular Structure & Function, Research Institute, Hospital for Sick Children, Toronto, Ontario, M5G 0A4, Canada; 2 Department of Biochemistry, University of Toronto, Toronto, Ontario, M5S 1A8, Canada; University of Pittsburgh, UNITED STATES

## Abstract

Accurate measurement of membrane protein stability—and particularly how it may vary as a result of disease-phenotypic mutations—ideally requires a denaturant that can unfold a membrane-embedded structure while leaving the solubilizing environment unaffected. The steric trap method fulfills this requirement by using monovalent streptavidin (mSA) molecules to unfold membrane proteins engineered with two spatially close biotin tags. Here we adapted this method to an 87-residue helix-loop-helix (hairpin) construct derived from helices 3 and 4 in the transmembrane domain of the human cystic fibrosis transmembrane conductance regulator (CFTR), wherein helix-helix tertiary interactions are anticipated to confer a portion of construct stability. The wild type CFTR TM3/4 hairpin construct was modified with two accessible biotin tags for mSA-induced unfolding, along with two helix-terminal pyrene labels to monitor loss of inter-helical contacts by pyrene excimer fluorescence. A series of eight constructs with biotin tags at varying distances from the helix-terminal pyrene labels were expressed, purified and labeled appropriately; all constructs exhibited largely helical circular dichroism spectra. We found that addition of mSA to an optimized construct in lipid vesicles led to a complete and reversible loss in pyrene excimer fluorescence and mSA binding, and hence hairpin unfolding—results further supported by SDS-PAGE visualization of mSA bound and unbound species. While some dimeric/oligomeric populations persist that may affect quantitation of the unfolding step, our characterization of the design yields a promising prototype of a future platform for the systematic study of membrane protein folding in a lipid bilayer environment.

## Introduction

Protein stability is the ultimate determinant of folding. In thermodynamic terms, protein stability can be quantified as a reversible change from a folded state to a fully unfolded state [1]—an energy difference that can vary significantly upon mutation of the protein sequence in a given environment. Stability analyses are performed routinely in the study of globular water-soluble proteins [2,3], where a sample protein solubilized in a native-like environment is treated with increasing levels of a denaturant, which could consist of chaotropic agents (urea, guanidinium chloride), harsh detergents (SDS), temperature (cold or heat), or external mechanical force. Unfolding can then be monitored through structural or functional loss until the protein has reached its unfolded state. Thermodynamically, this unfolding must be reversible and thus return the protein to its initial folded state upon removal of the denaturant.

However, this category of experiments is more complex in membrane proteins since additional components are required to create the membrane-like environment as the sample is solubilized. Indeed, no membrane-mimetic is insensitive to bulk denaturants, as they can alter the properties of detergent micelles and lipid bilayers used in protein folding studies [4–6]. Unfolding membrane proteins has been achieved with chaotropic agents [6,7] (urea/guanidinium HCl) or harsh detergents [8–10] (SDS), but these denaturants may entail confounding factors [6,11] in protein stability analysis in the membrane environment. In this context, recent efforts led to the development of the steric trap [12,13]—a method to unfold membrane proteins without affecting their membrane environment. Here, rather than bulk denaturants, monovalent streptavidin [14] (mSA) molecules are added to specifically bind covalently incorporated biotin tags that act as nano-mechanical ‘handles’. Once this binding is in place, these handles can sterically break protein contacts due to the high energy of streptavidin-biotin non-covalent interaction [15] without interacting with the hydrophobic environment embedding the membrane protein. Unfolding can be fully reversed by competition with excess free biotin, making the system thermodynamically valid for stability measurements. Following proof-of-principle with the globular protein dihydrofolate reductase [15], the Bowie group has elegantly applied this unfolding method to membrane proteins (GpA [12,13], diacylglycerol kinase [16], and bacteriorhodopsin [17]) in both detergent micelles and in lipid bilayers.

In the present work, we report the adaption of this method to the ‘hairpin’ system [18–20] in lipid bilayers, in which the hairpin consists of an 87-residue helix-loop-helix construct derived from transmembrane helices 3 and 4 of the human cystic fibrosis transmembrane conductance regulator (CFTR). Our principal goal with such a construct—essentially the simplest model of membrane-based tertiary contacts—was to design a simplified platform that would allow a systematic analysis of mutation impact—particularly where CF-phenotypic mutations are involved—on helix-helix interactions, and on overall hairpin stability. Accordingly, a series of hairpin constructs starting with the wild-type CFTR TM3/4 sequence were engineered with systematically inserted helix-terminal pyrene moieties as unfolding probes, and two biotin tags as the ‘handles’ for monovalent streptavidin. Using pyrene excimer fluorescence as a measure of inter-helical distance, these constructs were then exposed to streptavidin to evaluate their unfolding potential. Ultimately, an optimal TM3/4 design was determined for use in the measure of TM3/4 stability from binding curves built from mSA titrations. Overall, our results indicate that the steric trap is amenable to the TM3/4 hairpin system, while highlighting the variables that arise in the stability analysis of these constructs reconstituted in lipid bilayers.

## Materials and Methods

### Molecular engineering of biotin handles into CFTR TM3/4 constructs

**Fig 1:**
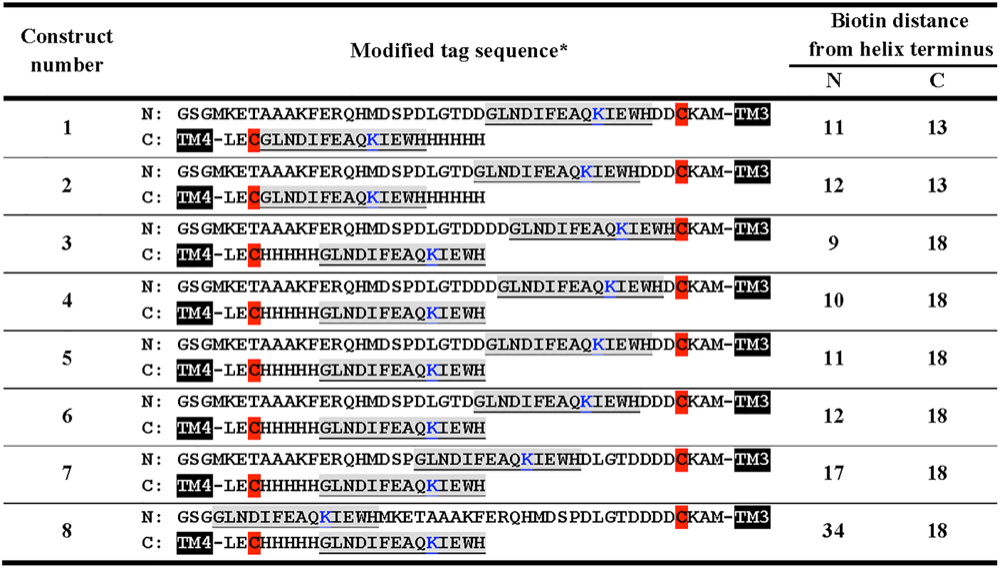
Human CFTR TM3/4 sequence designs with varying biotinylation site positions. *TM3/4–2C constructs were engineered to contain a biotinylation sequence (rendered in gray, with interactive Lys residue shown in blue) at various distances from each of the two helix-terminal Cys residues. The two Cys residues in each construct are highlighted in red. Only the tag sequences are shown; the solid TM3 and TM4 blocks indicate the adjacent transmembrane segments. For the full TM3/4 hairpin sequences, see the Materials and Methods section.

The sequence of the wild-type 87-residue hairpin construct used in the present work is GSGMKETAAAKFERQHMDSPDLGTDDDDCKAM^194^GLALAHFVWIAPLQVALLMGLIWELLQASAFAGLGFLIV-LALFQA-GLG^241^LECHHHHH, which embeds human CFTR residues 194-241 along with purification tags from the vector. Using the QuikChange Lightning site-directed mutagenesis kit (Agilent Technologies) biotinylation sites were inserted at varying distances from the N- and C-terminal ends of TM3 and TM4, respectively, into hairpin constructs already containing two helix-terminal cysteines for subsequent pyrene labeling [19]. The manufacturer’s protocol for nucleotide insertion was followed to first insert into TM3 the sequence 5’-GGCCTGAACGACATCTTCGAGGCTCAGAAAATCGAATGGCAC-3’, encoding for the 14-residue biotin acceptor sequence GLNDIFEAQKIEW where Lys_10_ is biotinylated [21]. This insertion was subsequently repeated on the TM4 segment terminus to yield constructs with two biotinylation sites. Varying primer targeting led to the preparation of a series of constructs with biotinylation sites at various distances from helix-terminal cysteine residues for pyrene labeling. Of the ten constructs obtained, eight could be expressed heterologously, purified, and biotin-labeled (Fig. 1).

### Expression and biotin labeling of CFTR TM3/4 hairpin constructs

**Fig 2:**
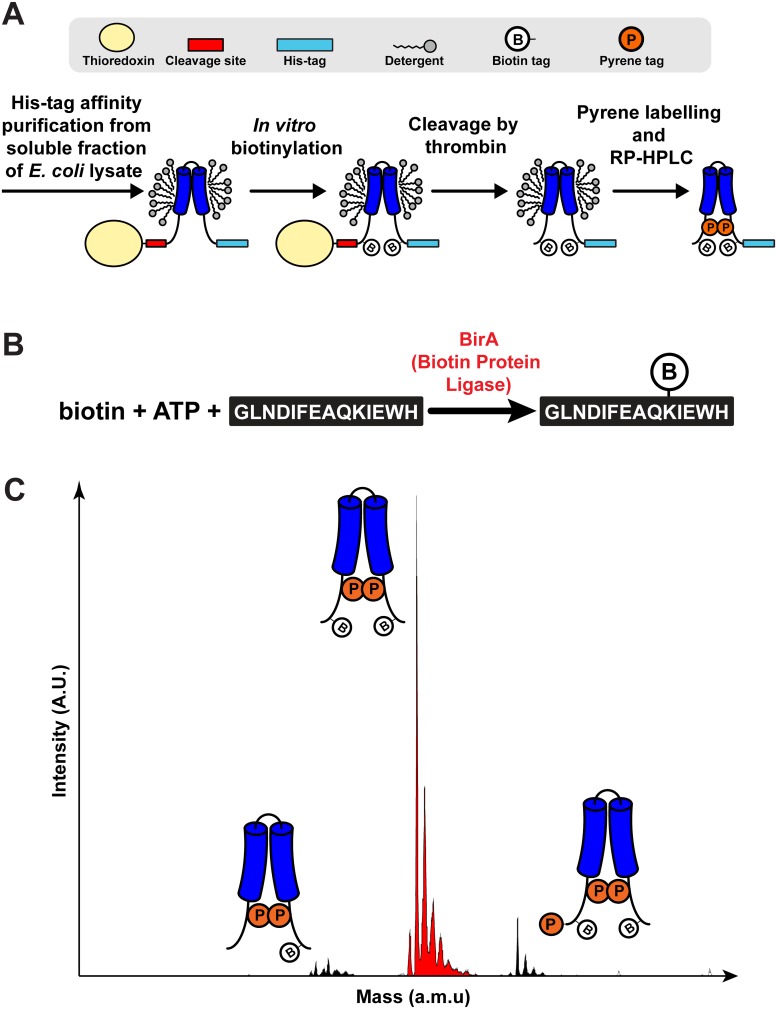
Preparation of unfolding-competent CFTR TM3/4 constructs. (**A**) Purification and labelling scheme to obtain TM3/4 constructs. Following His-tag affinity purification from the soluble fraction of *E*. *coli* lysates, constructs are biotinylated *in vitro* using the reaction shown in (**B**). Then, biotinylated constructs are cleaved by thrombin to remove the solubilizing thioredoxin moiety, and labeled with pyrene iodoacetamide at each of two cysteine residues. Constructs are purified from excess reagents, cleaved thioredoxin, and detergent, by RP-HPLC. (**B**) *In vitro* biotinylation reaction scheme using free biotin, ATP, and purified biotin protein ligase (BirA). This reaction targets Lys^10^ in the biotin acceptor peptide sequence [21] inserted in TM3/4 water-soluble region by site-directed mutagenesis (see Methods). (**C**) Typical mass spectrum for a purified CFTR TM3/4 construct, highlighting the predominance of a doubly biotinylated and pyrene-labeled version (*red*). Trace amounts of constructs with only one biotin tag or with an extra pyrene label are still present after purification (*black*).

CFTR TM3/4 constructs were expressed in *E*. *coli* BL21 (DE3) star cells (Life Technologies) and purified from the soluble fraction of bacterial lysates by His-tag affinity chromatography following previously published protocols [22] with modifications after protein elution (Fig. 2A). Thus, after pooling fractions from His-tag affinity chromatography, the thioredoxin tag was removed by addition of 20–30 units of Thrombin (Novagen) and incubation for 16 hours at room temperature with constant mixing. Samples were then dialyzed against 20 mM Tris, and 0.3% Triton X-100, pH 8, in dialysis bags with 10 kDa molecular weight cut-off (MWCO). *In vitro* biotinylation was then initiated by adding 5 mM magnesium acetate, 30 mM Tris, 5 mM adenosine triphosphate, 1 mM D-biotin, pH 8 and 500 μg of purified biotin protein ligase (produced and purified according to [23]). The reaction was maintained for 24 hours at room temperature with constant mixing (Fig. 2B). The modified samples were dialyzed against 0.1% Triton X-100, 20 mM Tris, 1.5 mM tris(2-carboxyethyl)phosphine (TCEP) at pH 7.5 in dialysis bags with 10 kDa MWCO. Then, 1 mM pyrene-iodoacetamide from a freshly prepared stock in dimethylformamide was added, and each sample was blanketed with nitrogen gas. The reaction was maintained for up to 36 hours and quenched for at least one hour with 10 mM β-mercaptoethanol. Labeled products were purified from reagents by reverse-phase high performance liquid chromatography (RP-HPLC) using the protocol described in [22]. Biotinylation and pyrene labeling was confirmed by mass spectrometry (Fig. 2C). Purified samples were frozen at -80°C and lyophilized to dryness for storage at -20°C.

### Production of monovalent streptavidin (mSA)

The procedure for the expression and purification of monovalent mSA mutants was adapted from protocols from the Ting [14] and the Bowie [12] labs. First, plasmids encoding ‘dead’ (mutant subunit lacking biotin affinity), S45A, and E44Q/S45A [the latter two ‘alive’ (biotin-interactive) subunits containing a C-terminal His-tag] were expressed in BL21 (DE3) star cells (Life Technologies) in Luria Broth medium with 4 hours induction with 0.5 mM IPTG at 37°C. Cells were harvested by centrifugation in a JLA 9.1 rotor (Beckman-Coulter) at 7500 rpm, 4°C for 10 minutes. Bacterial pellets were resuspended in 10 mL of 50 mM Tris and 1 mM phenylmethylsulfonyl fluoride at pH 8 per liter of initial culture. Cells were lysed by passing them 4–6 times through a homogenizer at 15 kPa. 1 mg/mL lysozyme as a powder (Sigma-Aldrich) and 25 units/mL of DNase I (Sigma-Aldrich, stock made in 50% glycerol, 50 mM Tris, 10 mM CaCl_2_ and 10 mM MgCl_2_, pH 7.5) was added to the lysate, supplementing the sample with 10 mM CaCl_2_, and incubated at room temperature for 15 minutes. Inclusion bodies were precipitated by centrifugation in a JA-20 rotor (Beckman-Coulter) at 20,000 rpm, 4°C for 15 minutes, and washed three times with the following cycle: resuspension using a tissue grinder with 10 mL/liter of initial culture of 50 mM Tris, 1.5 M NaCl, 1 mM phenylmethylsulfonyl fluoride, 1% Triton X-100, 25 units/mL DNase I, 10 mM CaCl_2_, pH 8; and centrifugation in a JA-20 rotor (Beckman-Coulter) at 20000 rpm, 4°C for 15 minutes. Then, inclusion bodies were washed once using the same procedure as above but without detergent or DNase I.

Inclusion bodies were solubilized with 4 mL of 6 M guanidine HCl, pH 2 per liter of initial culture. Insoluble aggregates were removed by centrifugation in a JLA-16.25 rotor (Beckman-Coulter) at 12000 rpm, 4°C for 5 min. Protein concentration in the supernatant was approximated using UV absorption at 280 nm and 3–4 parts dead subunit mixed to 1 part alive subunits. Tetrameric streptavidin was refolded by dropwise, rapid dilution in ice-cold 20 mM sodium phosphate, 200 mM NaCl, and 400 mM L-arginine, pH 7.5, yielding a 1/50 to 1/80 final dilution. The refolding reaction was then incubated with mixing for 1 hour at 4°C before removing aggregated proteins by centrifugation in a JLA 10.5 rotor (Beckman-Coulter) at max speed, 4°C for 1 hour. Supernatant with refolding product was then extracted with two successive ammonium sulfate precipitations: a first cut at 42.5% to precipitate contaminating proteins with 4 hours incubation at 4°C; and a second cut at 90% to precipitate tetrameric streptavidin with overnight incubation at 4°C. In both cases, the insoluble fraction was pelleted by centrifugation in a JLA 10.5 or JLA 9.1 rotor (Beckman-Coulter) at max speed, 4°C for 1 hour. Precipitated tetrameric streptavidin was then solubilized in 20 mL of 20 mM sodium phosphate and 200 mM NaCl, pH 7.5 per liter of initial culture. Samples were dialyzed against 20 mM sodium phosphate and 200 mM NaCl, pH 7.5 in dialysis bags with 10 kDa MWCO to remove excess ammonium sulfate salt and monovalent streptavidin was purified from multivalent species using His-tag affinity chromatography as in [12]. Following purification and dialysis against 50 mM NaCl, pH 9, 5 mg aliquots were lyophilized and stored at -20°C for future use.

### Reconstitution of TM3/4 constructs

Reconstitution of TM3/4 constructs in 1-palmitoyl-2-oleoyl-*sn*-glycero-3-phosphocholine (POPC) vesicles was performed as previously described [22] except for a change in the reconstitution buffer, where 50 mM sodium phosphate and 200 mM NaCl, pH 7.5. 2 3M protein was used. Lipid-only controls were also prepared following the same protocol but without using protein. Five different lipid/protein ratios were used for the reconstitution: 250, 500, 1000, 2000, and 3000.

Lyophilized mSA samples were solubilized in 50 mM sodium phosphate and 200 mM NaCl, pH 7.5 to a final concentration of about 5 mg/Mr. Samples were dialyzed against the same buffer to equilibrate samples in dialysis bags with 10 kDa MWCO. The mSA concentration was determined using UV absorption at 280 nm.

### Circular dichroism spectroscopy of TM3/4 constructs in lipid bilayers

TM3/4 construct 2 in lipid bilayers was analyzed by CD spectroscopy in 50 mM sodium phosphate and 200 mM NaCl, pH 7.5, following the same protocol as described in [22]. Sample spectra were corrected with a spectrum for lipid vesicles at the respective lipid concentration but without protein.

### Fluorescence-based monitoring of mSA titrations

mSA titrations were set up in a 96-well plate format in 50 mM sodium phosphate and 200 mM NaCl, pH 7.5, with triplicates of 2 μM TM3/4 construct 2 reconstituted in POPC bilayers with 0 to 15 μM mSA; and a single replicate for each mSA concentration with lipid vesicles but without protein. All samples had a final volume of 200 all. These conditions were repeated for each lipid/protein ratio tested (250, 500, 1000, 2000, 3000), and for each mSA mutant used (S45A and E44Q/S45A). Samples were allowed to equilibrate for 24 hr before measurement.

Pyrene fluorescence spectra were measured using a Spectra Mac Gemini fluorescent plate reader with excitation at 345 nm and emission scan from 380–550 nm at 2 nm resolution. Each spectrum was measured with 6 accumulations and medium sensitivity. Then, to measure reversibility of mSA-induced unfolding, 3.75 mM D-biotin was added to each well and samples were allowed to equilibrate for 24 hrs. Spectral measurements were repeated using the same parameters as above.

Pyrene fluorescence spectra were corrected with the spectrum measured with the respective mSA concentration and lipid concentration. To obtain excimer/monomer ratios (E/M), the area under the excimer peak (446–550 nm) was divided by the area under the monomer peaks (390–420 nm).

### mSA binding analysis by Western blot

24 pmoles of construct 2 in POPC bilayers (lipid/protein = 2000) with each mSA concentration before and after biotin treatment were prepared rapidly in 1X sample buffer without boiling. All samples were run on a 10% Tris-Glycine SDS-PAGE at 125 V, keeping the gel box on ice. PAGE was transferred onto a nitrocellulose membrane at 300 mA for 1 hour and membranes were blocked with 5% skim milk in Tris-buffered saline containing Tween 20 (TBS-T) overnight at 4°C. After washing with TBS-T, the membrane was treated with 1/5000 S-protein-HRP in TBS-T for 1 hour at room temperature. After 5 brief washes with TBS-T, the membrane was treated with the ECL Prime Western Blot Detection Kit (GE Healthcare) and an image of the membrane was acquired with a LICOR Odyssey instrument.

## Results

### Design and preparation of a helix-loop-helix membrane protein unfolding platform

**Fig 3:**
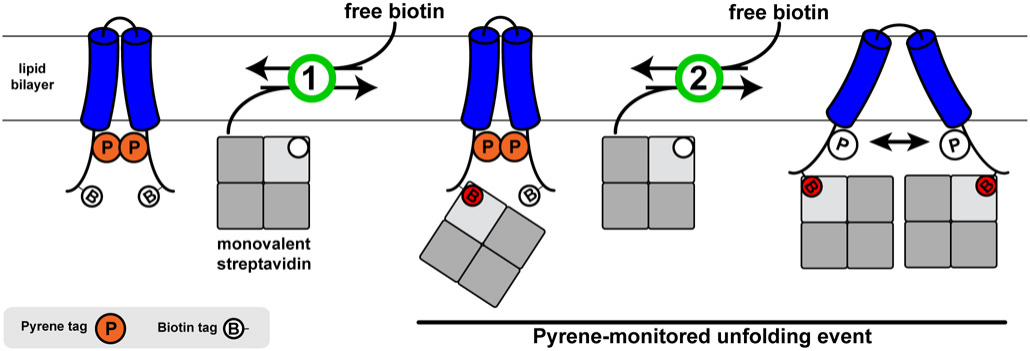
Membrane protein hairpin platform for folding studies. A CFTR TM3/4 variant containing two helix-terminal pyrene tags, along with two water-exposed biotin labels, can be unfolded while inserted in a lipid bilayer in a controlled manner using the high affinity biotin-streptavidin interaction with monovalent streptavidin (mSA). At low mSA concentrations, only one biotin tag is occupied (1), while increasing mSA concentration leads to steric unfolding of the hairpin (2) to allow for the second binding to occur. This process can be reversed by competition with excess free biotin. Using pyrene as a probe of interhelical distance, step 2 of this process is monitored experimentally.

We engineered CFTR TM3/4 hairpins with helix-terminal pyrene tags and two proximal biotin moieties that would be accessible to mSA in a preparation embedded in unilamellar lipid vesicles. In order to obtain double pyrene-labeled as well as doubly biotinylated constructs in a controlled and efficient manner, constructs were biotinylated enzymatically at Lys_10_ of two engineered 14-residue biotinylation acceptor peptides at close to 100% efficiency (Fig. 2A and B). Pyrene labeling could then be done chemically by targeting the cysteine residues a few residues away from the predicted [19] helix termini with high efficiency. In order to generate a series of potential platform constructs, the distance between the biotinylation sequences and the pyrene moieties was systematically varied, thus yielding a range of sites for steric hindrance that may induce unfolding (Fig. 3). Among ten designs tested, eight constructs (Fig. 1) could be expressed at viable levels in *E*. *coli*, purified, and labeled as per the above protocol (Fig. 2A). In all cases, the final pure constructs contained only minimal amounts of side-products, *viz*., the single-biotinylated and triple pyrene-labeled side-labeling products (Fig. 2C).

With this design—assuming that an initial binding of mSA to one biotin tag does not affect structure (Fig. 3, step 1)—binding of a second mSA molecule would, in principle, require unfolding of TM3/4 tertiary contacts due to steric hindrance (Fig. 3, step 2). Thus, use of pyrene excimer fluorescence as a probe of inter-helical distance [24] allows the readout of only the unfolding event, *i*.*e*. binding of the second mSA molecule. By using mutant mSA with lower and reversible affinity to biotin[12] (K_d, biotin_ ≈ 10^-9^ M) relative to the strong virtually irreversible binding of WT mSA (K_d, biotin_ = 10^-14^ M), TM3/4 hairpins can then be refolded by competition with excess free biotin.

### Search for an optimal TM3/4 platform construct

**Fig 4:**
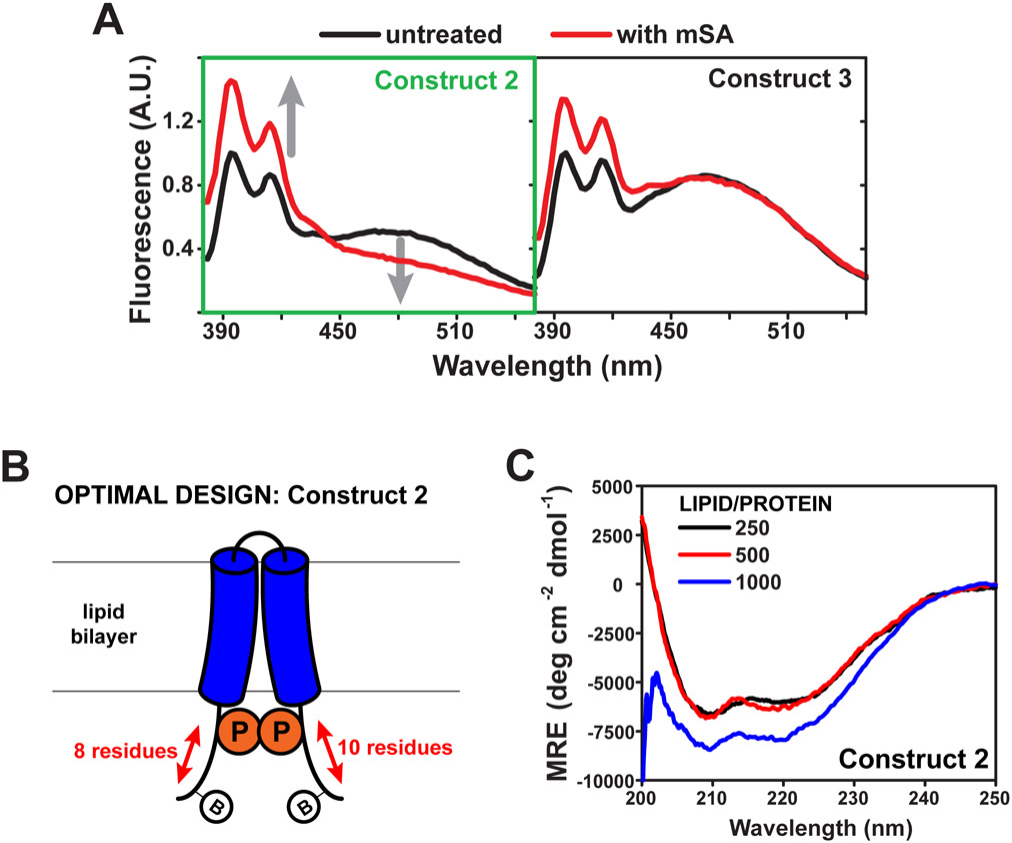
Assay for an optimal TM3/4 unfolding platform. (**A**) Pyrene fluorescence spectra in the absence or presence of 20 μM S45A mSA for two TM3/4 constructs designed, expressed, purified, and reconstituted in POPC bilayers (lipid/protein = 2000), 50 mM sodium phosphate pH 7.5, and 200 mM NaCl. Peaks between 380–440 nm are due to emission of monomeric pyrene while the broad peak centered around 480 nm is indicative of pyrene excimers, and thus of proximity of the two pyrene labels. Upon addition of mSA, construct 2 (highlighted in green) exhibits a drop in excimer peak with a concomitant increase in monomer intensity. (**B**) Schematic of the optimal construct selected for the unfolding platform. Biotin tags are relatively close to N- and C- terminal pyrene labels, 8 and 10 residues away respectively. (**C**) CD spectra of construct 2 at various lipid/protein ratios in 50 mM sodium phosphate pH 7.5, and 200 mM NaCl. Higher ratios tested did not yield reliable spectra due high light scattering by high lipid concentrations. At all ratios tested, construct 2 is helical. MRE = molar residue ellipticity. CD experiments were performed in triplicate.

With purified constructs reconstituted into large unilamellar vesicles, we evaluated the extent of pyrene fluorescence spectral change as a proxy for unfolding, assaying for an almost complete loss in excimer fluorescence (broad peak around 480 nm) paired with an increase in monomer intensity (double peak between 390 and 430 nm) upon addition of mSA (Fig. 4A). In this approach, constructs 3–8 exhibited minimal excimer fluorescence intensity loss and significant remaining excimer fluorescence, along with some increase in monomer fluorescence. However, constructs 1 and 2 fit well with the predicted model of excimer loss paired to monomer increase.

Since construct 2 showed the most complete excimer loss, and could be produced in much larger quantities in *E*. *coli*, it was selected to test the platform. This TM3/4 design exhibits biotin tags 8 and 10 residues away from the N- and C-terminus of TM helix ends, respectively (Fig. 1; Fig. 4B). We confirmed that this construct adopts a helical conformation in lipid bilayers by CD spectroscopy-at three different lipid/protein ratios (Fig. 4C), shown with spectral double minima around 208 and 222 nm from low to high lipid/protein ratios.

### Reversible unfolding of construct 2

**Fig 5:**
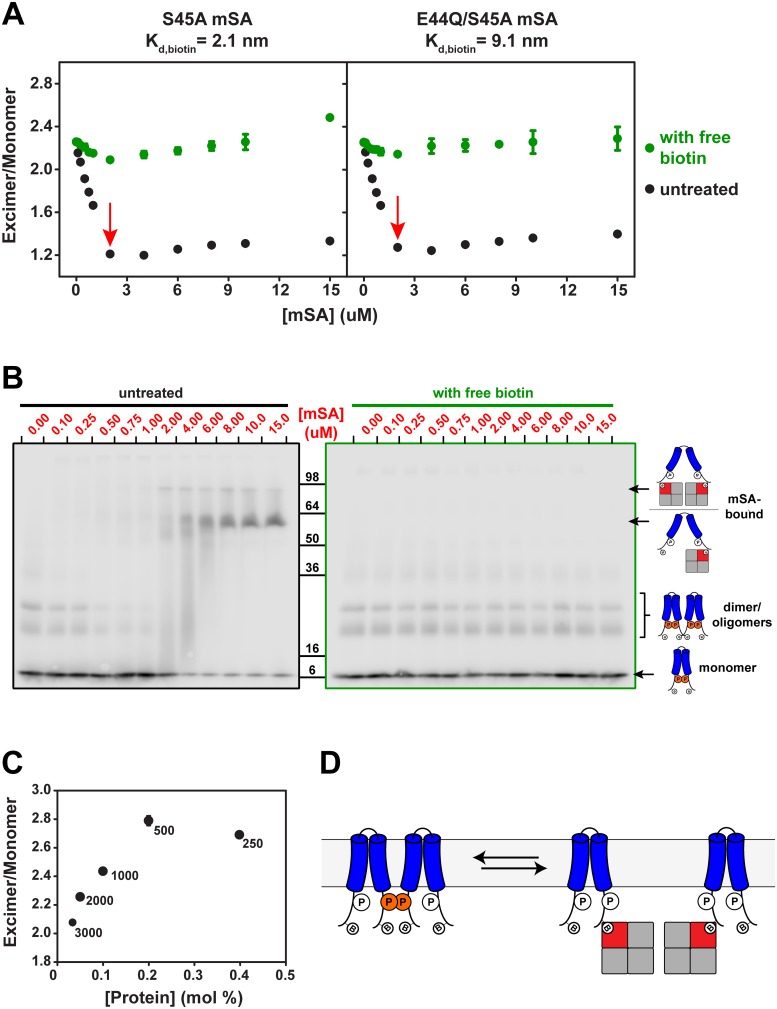
Unfolding and refolding TM3/4 Construct 2. (**A**) Monitoring the E/M ratio as a function of two mSA mutants with varying affinity towards biotin. Titrations are performed in 50 mM sodium phosphate pH 7.5, and 200 mM NaCl with a lipid/protein = 2000. With both mSA mutants, the E/M ratio drops by addition of mSA, indicating a loss in pyrene-pyrene contacts and therefore unfolding. The change in E/M ratio saturates upon reaching the total concentration of construct 2 (*i*.*e*. 2 μM, *red arrows*). On the other hand, addition of excess 3.75 mM free biotin samples after measurement leads to an almost complete negation of mSA effects on the E/M ratio, highlighting the reversibility of the unfolding reaction. Points shown are the average of three independent experiments; error bars indicate +/- standard deviation. (**B**) Effect of E44Q/S45A mSA titration monitored by separation of TM3/4 populations on SDS-PAGE. Detection is specific towards TM3/4 constructs using Western blotting against an S-tag. The mSA-biotin interaction is SDS-resistant, since samples were not boiled and SDS-PAGE was run on ice. On the left panel, small oligomers and the monomeric form of TM3/4 are observed without mSA and disappear as it is titrated, while mSA-bound TM3/4 populations appear concomitantly. In the presence of excess biotin (right panel), no mSA-bound TM3/4 populations exist. (**C**) E/M ratios for five different lipid/protein ratios, without mSA present. As the lipid/protein ratio increases, the effective protein concentration in lipid bilayers decreases. **(D)** Unfolding of TM3/4 quaternary structure. In this model, loss of pyrene excimer fluorescence is due to mSA unfolding of inter-hairpin contacts.

With a TM3/4 construct exhibiting optimal expression, pyrene fluorescence response to mSA, and helicity in lipid bilayers, we undertook to characterize the unfolding of this hairpin with full mSA titrations. Thus, we recorded the excimer/monomer (E/M) ratio for increasing mSA concentration as a measure of the relative loss in excimer peak area relative to monomer peak increase (Fig. 5A). Using mSA mutants with varying affinities for free biotin, we could observe in both cases a binding curve with two linear phases: an initial sharp decrease in E/M ratio followed by an almost flat saturation of the readout. Thus, we could not fit the typical binding curve expected for a single binding site, *i*.*e*., the unfolding event quantitated with the E/M ratio readout (Fig. 3, step 2). However, we noted that the inflection point between the two linear phases occurs at the total protein concentration used in the assay, perhaps suggesting that the affinity of these mSA mutants to the biotin tags is too strong relative to the stability of inter-helical interactions to provide a complete binding curve.

After measuring the effect at each mSA concentration, we added excess biotin to allow for TM3/4 refolding (Fig. 5A). We found that E/M ratios returned almost completely to values without mSA present, thus exhibiting minimal dependence on mSA present. This observation is strong evidence for the reversible unfolding/refolding of construct 2 in lipid bilayers. Yet, remnants of the biphasic relationship among E/M ratios still appear with biotin present, particularly for the case of the higher affinity S45A mSA mutant. This may indicate that the excess 3.75 mM free biotin is not enough to fully reverse the binding in the overnight time course of these experiments.

We could also assess mSA binding to TM3/4 constructs by separating bound and unbound populations of hairpins on SDS-PAGE with Western blotting against the S-tag present in our construct (Fig. 5B). The tetrameric mSA and its binding to biotin tags are resistant to unfolding by SDS if samples are not boiled and the gel is run at 4°C. Thus, we found that mSA-bound hairpin populations appeared as low as 0.75 μM mSA (Fig. 5B, left panel), which matches the midpoint of the first linear drop of the binding curve (Fig. 5A). However, small oligomers of our hairpin constructs were visible on SDS-PAGE, indicating either that these oligomers exist in our lipid bilayer system, and/or that they are an artifact of SDS solubilization. Relative to the oligomeric populations on SDS-PAGE, the monomeric species did not completely disappear at saturating mSA concentrations (Fig. 5B, left panel), indicating that a fraction of biotin tags is not available to bind mSA. Interestingly, addition of excess free biotin to E44Q/S45A mSA-treated construct 2 samples fully abrogates the formation of mSA-bound populations (Fig. 5B, right panel), thus matching the E/M ratio-based binding curve (Fig. 5A).

We investigated the oligomerization concerns mentioned above by testing construct 2 unfolding in increasing lipid/protein ratios, thus decreasing effective protein concentration in the lipid bilayer. Varying such a parameter allows us to minimize the effects of potential oligomerization of our samples, thus decreasing the impact of hypothetical dimer unfolding monitored by our pyrene-based assay. Indeed, we noticed that the initial E/M ratio in these titrations increased with effective protein concentration in lipid bilayers (Fig. 5C), showing that dimeric/oligomeric populations could account for some of the initial pyrene excimer signal measured (Fig. 5D).

## Discussion

With the success of the steric trap method in evaluating the stability of quaternary contacts [12,13,15] and tertiary contacts [17], we undertook to build a platform that would enable the systematic evaluation of mutation impact on tertiary structure stability—in the present instance, helix-helix interactions in a membrane protein hairpin construct. With a large library of CF-phenotypic and artificial mutations in CFTR TM3/4 in hand [18,20,25–27], such a platform would allow for a quantitative analysis of the structural impact of these mutants.

### Effects of mSA addition to CFTR TM3/4 construct 2 structure in a lipid vesicle system

Using our designed TM3/4 construct, we found that significant changes in pyrene excimer fluorescence occur upon mSA addition. Knowing that TM3/4 hairpins span the lipid bilayer in a helical conformation [22], these molecular tweezers must disrupt inter-helical contacts to create an almost complete loss in pyrene excimer fluorescence (Fig. 4A). Thus, in binding the engineered biotin tags in the hairpin construct, mSA effectively forces open the membrane-embedded TM3/4 helices to prevent unfavorable steric clashes between the large streptavidin tetramers tightly bound to biotin tags (Fig. 3).

A further factor that could affect unfolding is the orientation of insertion of the TM3/4 construct 2 in lipid vesicles as it would impact the availability of biotin tags to external mSA molecules. The existence of this parameter is exemplified by the presence of unbound monomers on SDS-PAGE at concentrations past the saturation of E/M signal in mSA binding curves (Fig. 5). Indeed, only hairpins inserted to have soluble tag regions on the outside of vesicles can be bound by mSA and unfolded. Fortunately, our proteoliposome reconstitution protocol yields mostly such an orientation considering the very small fraction of unbound hairpin on SDS-PAGE (Fig. 5B, left panel). Yet, such a factor would require normalization by quantifying the concentration of exposed biotin tags relative to total protein present, perhaps with fluorescent mSA probes.

There remains the possibility that streptavidin interacts with the fluorophore in a weak, indirect manner, albeit this protein is well-known to be highly specific to biotin, and in such a scenario, we expect only transient interactions to affect the pyrene label. Rotation of pyrene labels could affect the signal measured, so these tags were intentionally placed in a region of sequence that is expected to be unstructured; thus, these tags will, in principle, be flexible. Nevertheless, the fact that we can measure pyrene excimer fluorescence indicates that the pyrene tags are indeed interacting.

While we noted that a lower helical signal intensity for construct 2 (Fig. 3C) relative to previous observations for WT TM3/4 in lipid bilayers [22], this discrepancy should be due to the larger tag region in construct 2 vs. WT TM3/4—the former containing two long biotinylation tags that may not adopt any specific secondary structure. As such, it is unlikely that the sequence additions/substitutions made to hairpin segments external to the bilayer will alter the initial TM3/4 helical content/interactions.

Given that we are dealing with large hydrophobic molecules, some degree of non-specific pyrene-pyrene interaction may be a prevalent side effect that must generally be confronted in hairpin systems and related model designs. Thus, the most obvious confounding variable in the present analysis is the potential presence of dimeric (or higher oligomer) hairpin species in our system, as evidenced in the SDS-PAGE results (Fig. 5B), and the excimer fluorescence dependence on effective protein concentration (Fig. 5C). As such, we must consider that at least a fraction of TM3/4 hairpin dimers in a given sample unfolds upon mSA addition (Fig. 5D)—an event that could explain some of the pyrene excimer fluorescence loss in the mSA binding curves. Indeed, it cannot be unequivocally discerned whether E/M loss is paired, at least in part, with dimer or monomer binding to mSA in the SDS-PAGE results (Fig. 5B, left panel). Ultimately, a complete binding curve [12] without biphasic behavior would be required to separate any extant effects of monomer vs. dimer unfolding.

## Conclusion

We have shown that an optimized wild type CFTR TM3/4 hairpin can be unfolded by mSA titration in a reversible manner while embedded in a lipid vesicle system—a construct design that allows for the systematic and precise evaluation of the effects of various mutations on inter-helical interactions, particularly those that evoke human disease. Overall, we expect that our platform yields a global unfolding output, as streptavidin molecules are significantly larger than the TM3/4 hairpins. While experiments performed with a range of hairpin concentrations, and/or with further streptavidin mutants, would further refine the platform, the present results do portend the general utility of the method for assessing quantitation of TM helix-helix association energetics. Indeed, several mSA mutants with lower affinity than S45A and E44Q/S45A are available to tune the strength of steric unfolding. Such a modifiable platform will ultimately allow for extensive studies to define key sequence factors in membrane protein folding.
